# Medical Association of Georgia

**Published:** 1877-04

**Authors:** 


Medical Association of Georgia.—The annual meeting of this body, for the year 1877, will be held in Macon on the third Wednesday (18th) of April, inst., and continue three days.

The location selected being convenient to a large number of physicians, a full attendance and quite an accession of new members may be expected. Doubtless the profession of Macon will strive to make the occasion an enjoyable one, in addition to the great feast these annual scientific discussions afford.

